# Effects of Transcutaneous Vagus Nerve Stimulation (tVNS) on the P300 and Alpha-Amylase Level: A Pilot Study

**DOI:** 10.3389/fnhum.2018.00202

**Published:** 2018-06-21

**Authors:** Carlos Ventura-Bort, Janine Wirkner, Hannah Genheimer, Julia Wendt, Alfons O. Hamm, Mathias Weymar

**Affiliations:** ^1^Department of Psychology, University of Potsdam, Potsdam, Germany; ^2^Department of Psychology, University of Greifswald, Greifswald, Germany; ^3^Department of Psychology, University of Würzburg, Würzburg, Germany

**Keywords:** EEG, P300, tVNS, norepinephrine, locus coeruleus, salivary alpha-amylase

## Abstract

Recent research suggests that the P3b may be closely related to the activation of the locus coeruleus-norepinephrine (LC-NE) system. To further study the potential association, we applied a novel technique, the non-invasive transcutaneous vagus nerve stimulation (tVNS), which is speculated to increase noradrenaline levels. Using a within-subject cross-over design, 20 healthy participants received continuous tVNS and sham stimulation on two consecutive days (stimulation counterbalanced across participants) while performing a visual oddball task. During stimulation, oval non-targets (standard), normal-head (easy) and rotated-head (difficult) targets, as well as novel stimuli (scenes) were presented. As an indirect marker of noradrenergic activation we also collected salivary alpha-amylase (sAA) before and after stimulation. Results showed larger P3b amplitudes for target, relative to standard stimuli, irrespective of stimulation condition. Exploratory *post hoc* analyses, however, revealed that, in comparison to standard stimuli, easy (but not difficult) targets produced larger P3b (but not P3a) amplitudes during active tVNS, compared to sham stimulation. For sAA levels, although main analyses did not show differential effects of stimulation, direct testing revealed that tVNS (but not sham stimulation) increased sAA levels after stimulation. Additionally, larger differences between tVNS and sham stimulation in P3b magnitudes for easy targets were associated with larger increase in sAA levels after tVNS, but not after sham stimulation. Despite preliminary evidence for a modulatory influence of tVNS on the P3b, which may be partly mediated by activation of the noradrenergic system, additional research in this field is clearly warranted. Future studies need to clarify whether tVNS also facilitates other processes, such as learning and memory, and whether tVNS can be used as therapeutic tool.

## Introduction

Since its discovery (Desmedt et al., 1965; Sutton et al., 1965), the P300 has been one of the most studied event-related potentials (ERPs) associated with psychological processes (Nieuwenhuis et al., 2005; Polich, 2007; Duncan et al., 2009). The P300, or P3, is a scalp-recorded ERP characterized by a positive deflection with maximal amplitude over centro-parietal regions between 300 ms and 600 ms after stimulus onset. The P3 is modulated by a variety of psychological states such as the tonic arousal level (for review, see Polich and Kok, 1995), the attributed relevance of the evoking stimulus (Ritter et al., 1972), the subjective probability of its appearance (Gonsalvez and Polich, 2002; Croft et al., 2003), and the cognitive resources engaged in stimulus processing (Donchin and Cohen, 1967; Hillyard et al., 1973; Duncan-Johnson and Donchin, 1977). However, the P3 seems to be insensitive to the stimulus modality (Johnson, 1993), suggesting that P3 activity reflects cognitive rather than perceptual processing.

The P3 has been detected across a large number of tasks such as Go/No-Go (Albert et al., 2013), flanker (Clayson and Larson, 2011), or passive picture viewing tasks (Keil et al., 2002) and most reliably in oddball paradigms (Polich, 2007). Here, the presentation of a chain of frequent, irrelevant (i.e., standard) stimuli is intermixed with the presentation of non-frequent, relevant (i.e., target) events and participants are instructed to respond mentally or overtly to the target stimulus and not respond otherwise. This paradigm typically elicits an increased P3 amplitude to target compared to standard stimuli. Additionally, novel and highly deviant stimuli are also processed as relevant events. However, the presentation of non-frequent, novel stimuli evokes a P3, known as the P3a, with a more frontal-central distribution than the central-parietal P3 elicited by targets, also named P3b (Polich, 2007).

Despite their similarities, the P3a and P3b have been defined as two functionally and anatomically independent ERP components (Spencer et al., 1999; Polich and Comerchero, 2003; Polich, 2007; Brown et al., 2015). The P3a has been related to the attentional deployment during novelty processing (Polich, 2007) and working memory, and to activity of the prefrontal cortex (McCarthy and Wood, 1987; Potts et al., 1996; McCarthy et al., 1997). The P3b has been associated with deeper stimulus-evaluation mechanisms (Kutas et al., 1977; Duncan-Johnson, 1981; McCarthy and Donchin, 1981; Nieuwenhuis et al., 2005) for instance during decision-making and memory processes (Donchin and Coles, 1988; Polich, 2007), and has been related to activity in parieto-temporal cortices (Kiss et al., 1989; Smith et al., 1990; Halgren et al., 1995; Strange and Dolan, 2007). Some studies using intra-cranial EEG or simultaneous EEG-fMRI suggested that the P3b depends on widespread cortical (e.g., frontal and orbitofrontal gyrus), subcortical (e.g., hippocampus, amygdala, thalamus) and brain stem regions (Halgren et al., 1980; Yingling and Hosobuchi, 1984; McCarthy et al., 1989; Smith et al., 1990; Walz et al., 2013). Taken together, this evidence suggests that the neural mechanisms underlying the processing of target and novel events does not exclusively reflect the activity of specific brain regions, but rather the engagement of a broad neural network that involves several brain areas (Pineda et al., 1989), probably orchestrated by distinct neurotransmitter systems (Nieuwenhuis et al., 2005; Polich, 2007).

Indeed, some studies suggest that the P3a seems to be governed by the dopamine (DA) system (for a review see Polich, 2007), whereas the P3b seems to be modulated by the phasic response of the locus coeruleus-Norepinephrine (LC-NE) system (for reviews, see Nieuwenhuis et al., 2005; Polich, 2007). For instance, Poceta et al. (2006) examined the P3a and P3b in patients with disorders associated with dopaminergic deficits of different severity such as restless legs syndrome (moderate severity) and Parkinson’s Disease (PD; high severity), using a three-stimulus oddball task. The authors observed that the P3a amplitude, but not the P3b, linearly decreased with the severity of the disorder. In line, Solís-Vivanco et al. (2015) observed that patients with PD showed a reduced P3a compared to healthy controls, which was also associated with the duration of the disorder. Evidence for the involvement of the dopaminergic system on P3a amplitudes also comes from genetic studies showing that polymorphisms associated with larger phasic DA release generated larger P3a, but not P3b amplitudes (Marco-Pallarés et al., 2010; see also, Strobel et al., 2004; Heitland et al., 2013).

Although the dopaminergic system seems to be tightly related to the generation of the P3a, the relation to, and the function of the norepinephrine system for the P3b is less clear. Some studies found that pharmacologically reduced NE levels (via intake of clonidine or propranolol)—compared to placebo—produced smaller P3b amplitudes (Duncan and Kaye, 1987; Joseph and Sitaram, 1989; Halliday et al., 1994; Lovelace et al., 1996), while others found increased P3b amplitudes (Brown et al., 2015) that also varied with anxiety (de Rover et al., 2015), or no changes following noradrenergic modulation (Lovelace et al., 1996).

The main goal of the present study was to apply a novel technique, the so-called transcutaneous vagus nerve stimulation (tVNS), to further test the relation between the noradrenergic system and the P3b. Animal research has shown that direct stimulation of the vagus nerve afferents enhances NE release in the brain via LC brain stem activation (Dorr and Debonnel, 2006; Raedt et al., 2011). Unlike direct vagus nerve stimulation, tVNS is a new method that allows the non-invasive stimulation of the vagus nerve in humans without using implanted electrodes (Van Leusden et al., 2015; Yuan and Silberstein, 2016). Specifically, tVNS acts upon the auricular branch of the vagus nerve (ABVN) located between the tragus and the entry of the acoustic meatus (Kreuzer et al., 2012). Animal research has shown that the stimulation of the ABVN reaches the brain through direct projections to the nucleus of the solitary tract (NST; Nomura and Mizuno, 1984; He et al., 2013; Liu et al., 2014) and the LC (Van Bockstaele et al., 1999), which was also confirmed by recent human neuroimaging studies using non-invasive vagal stimulation (Dietrich et al., 2008; Kraus et al., 2013; Frangos et al., 2015). However, despite evidence for tVNS induced activation of this brainstem circuitry (e.g., LC), no data exist indicating that non-invasive VNS is related to noradrenergic activation in humans. We therefore tested the effects of tVNS on salivary alpha-amylase (sAA) levels, which is assumed to be an indirect marker for central NE system activation (Chatterton et al., 1996; Warren et al., 2017).

In a within subject cross-over design, participants performed a visual three-stimulus, novelty oddball task on two consecutive days, in which tVNS and sham stimulation were alternately administered. SAA levels were obtained before and after stimulation. Following the assumption that tVNS activates the noradrenergic system in humans, we predicted that tVNS, in contrast to sham stimulation, would result in greater increase of sAA levels. Furthermore, following the hypothesis that the LC-NE system modulates the P3b, we expected to find larger P3b amplitudes in response to targets following tVNS compared to sham stimulation. Because changes in P3b amplitudes (tVNS vs. sham stimulation) and in sAA levels (pre vs. post) are expected to be influenced by the potentiation of the LC-NE system, a positive relationship between both measures was predicted, particularly for the tVNS condition. Finally, we did not expect any tVNS effects on the P3a.

## Materials and Methods

### Participants

A total of 21 German-speaking students from the University of Greifswald (18 female; *M*_age_ = 20.3 years, SD = 1.4 years) participated for course credits or financial compensation. Each individual provided written informed consent for a protocol approved by the Review Board of the German Psychological Society and in accordance with the Declaration of Helsinki. All participants had normal or corrected-to-normal vision and all except one participant claimed right-handedness. Prior to the first session, participants were phone-screened and invited to participate when passing the following exclusion criteria: neurological or mental disorders, brain surgery, undergoing chronic or acute medication, pregnancy, history of migraine and/or epilepsy, heart-related diseases, metal implants in the face or brain, implants or physical alterations in the ear. Due to bad performance in the oddball task (i.e., no response given to targets), one participant was excluded from the analyses.

### Apparatus and Procedure

In the current study, a randomized, single-blinded, tVNS-sham, 2-day, within-subject, cross-over design was used. In each of the experimental sessions, participants alternately received either tVNS or sham stimulation.

Both sessions followed the identical protocol. Before undergoing stimulation, heart rate, blood pressure and sAA levels were measured (pre) while participants seated relaxed in the experimental room. Afterwards, the stimulation electrodes were applied to the left ear and the intensity was adjusted. In order to individually regulate the stimulation intensity, participants received increasing and decreasing series of 10-s stimulation trials, and rated the subjective sensation of the stimulation on a 11-point scale, ranging from nothing (0), light tingling (3), strong tingling (6), to painful (10). The increasing series of trials started from an intensity of 0.1 mA and increased 0.1 mA on a trial by trial basis until participants reported a “tingling” sensation of 9. Before starting the decreasing series, the same intensity was repeated and then reduced trial by trial in 0.1 mA steps until a subjective sensation of 6 or below was experienced. This procedure was repeated a second time. The final stimulation intensity used for the experimental procedure was calculated based on the average of the four intensities rated as 8 (i.e., 2 from increasing and 2 from decreasing series).

Then, the electroencephalography (EEG) net was applied and participants performed two experimental tasks: a novelty oddball task (Venables et al., 2011) which lasted 28 min, followed by a number version of the Simon task (Fischer et al., 2008, 2015), lasting 7 min. The results of the Simon task are reported elsewhere (Fischer et al., 2018).

After both experimental tasks, the EEG net and the stimulation electrodes were removed and heart rate, blood pressure, and sAA were measured again (post). Finally, participants were asked to report, on a seven-point scale (1 being “nothing” and 7 being “very much”), how much they experienced the following symptoms during the stimulation: headache, nausea, dizziness, neck pain, muscle contractions in the neck, stinging sensations under the electrodes, skin irritation in the ear, fluctuation in concentration or feelings and unpleasant feelings.

### Transcutaneous Vagus Nerve Stimulation

The tVNS stimulator consisted of two titan electrodes attached to a mount, which was located in the left auricle and wired to the stimulation unit (CMO2, Cerbomed, Erlangen, Germany). In the tVNS condition, the stimulator was placed in the left cymba conchae, an area innervated exclusively by the ABVN (Peuker and Filler, 2002; Ellrich, 2011). Alike previous studies using tVNS (e.g., Kraus et al., 2007; Steenbergen et al., 2015), in the sham condition, the electrodes were positioned in the center of the left ear lobe, an area known to be free of vagal innervation (Peuker and Filler, 2002; Ellrich, 2011). To ensure stimulation over the entire oddball task, the stimulation was delivered continuously with a pulse width of 200–300 μs at 25 Hz. Of note, this procedure of continuous stimulation differs to other stimulation protocols applying a 30 s ON and 30 s OFF procedure. The ABVN is related to touch sensation. Therefore, to ensure its activation, the stimulus intensity of the tVNS was set to be perceived (but with no discomfort). Thus, the tVNS was adjusted above the detection threshold and below the pain threshold (Ellrich, 2011). The average stimulation intensity for both conditions were as follows: 1.3 mA (0.4–3.3 mA) for active and 1.49 mA (0.6 mA −4.8 mA) for sham condition. The stimulation intensity did not differ between both conditions (*t*_(19)_ = 1.23, *p* = 0.23, *d* = 0.27). The stimulation was administered continuously during both experimental tasks for about 35 min.

Because the right vagal nerve sends efferent projections to the heart, the stimulation in the current study was always applied to the left ear to avoid the possibility of cardiac side effects. Recent studies, however, showed no side effects of right tVNS on cardiac activity in healthy participants (Kreuzer et al., 2012).

### Oddball Task

Participants performed a modified version of the rotated-heads oddball task (Begleiter et al., 1984) in which the non-target (standard) and “target” stimulus categories were complemented with a third novel category (Venables et al., 2011). The standard stimulus was a plain oval presented on 70% of the trials (*n* = 168). The target stimulus was a schematic head formed by the oval with a nose and an ear (15% of all trials; *n* = 36) and participants had to indicate whether the ear was presented on the left or on the right side of the nose by pressing the left or right button on a response-pad. In half of the target trials (*n* = 18), the nose was upwards (easy condition), and thus, the ear side matched with the side of screen. In the other half of the trials (*n* = 18), the nose was pointing down (difficult condition), which requires mental rotation of the head to recognize. The novel stimuli consisted of 36 emotional images presented once (15% of all trials). In total, 72 images (24 pleasant, 24 neutral and 24 unpleasant) were used, selected from the International Affective Picture System (IAPS; Lang et al., 2008). The images were divided into two sets carefully matched for emotional and physical attributes. Both sets were alternated between sessions. Before starting the experimental phase, participants performed 20 practice trials (including target, 50% and standard stimuli).

For the experiment, participants were seated in a comfortable chair in a dimly lit room, at a distance of 150 cm from a 17-inch monitor. Every stimulus was displayed on a dark gray, rectangular frame over a black background. The frame size extended to a visual angle of about 5° × 6.67°, vertically and horizontally, respectively. The plain oval and head stimuli were displayed within the frame at a visual angle of 3.50° × 3.75°, and the emotional pictures extended to the size of the frame. On each trial the stimulus was presented for 100 ms each, with a variable inter-trial interval between 6.5 s and 8 s. The stimuli were randomly presented with the restriction of not more than two non-frequent stimuli presented consecutively. Stimulus presentation and data recording were controlled by Presentation (Version 16.5; Neurobehavioral Systems Inc., Albany, CA, USA).

### Autonomic Measures

To evaluate the effects of stimulation on autonomic reactivity, we measured heart rate and blood pressure (systolic and diastolic) prior to the stimulation (pre), and after both experimental tasks (post). Heart rate was measured manually from the wrist of the left hand and blood pressure was assessed with an upper arm cuff placed on the left arm, using the Riva-Rocci method. In addition, sAA was also collected as an indirect marker of endogenous noradrenergic activation (Chatterton et al., 1996; Warren et al., 2017). sAA levels were collected out of saliva samples using regular cotton Salivette sampling devices (Sarstedt, Nümbrecht, Germany). Participants were instructed to gently chew the swab in their mouths for 60 s. After removal, saliva samples were stored at −20°C. Analyses were performed by the Dresden LabService GmbH^1^ (Thoma et al., 2012) using an enzyme kinetic method. Due to technical issues, alpha amylase levels from two participants had to be excluded from the analyses.

### Electrophysiological Recording

EEG signals were recorded continuously from 257 electrodes using an Electrical Geodesics (EGI) high-density EEG system with NetStation software on a Macintosh computer. The EEG recording was digitized at a rate of 250 Hz, using vertex sensor (Cz) as recording reference. Scalp impedance for each sensor was kept below 30 kΩ. All channels were band-pass filtered online from 0.1 Hz to 100 Hz. Stimulus-synchronized epochs were extracted from 200 ms before to 1200 ms after stimulus presentation and then submitted to the procedure proposed by Junghöfer et al. (2000), as implemented in the EMEGS software provided by Peyk et al. (2011). This procedure includes low-pass filtering (20 Hz), artifact detection, sensor interpolation, baseline correction (i.e., 200 ms prior to stimulus presentation) and conversion to the average reference (Junghöfer et al., 2000). The MATLAB-based toolbox BioSig (Vidaurre et al., 2011) was used for eye movement and blink artifacts corrections of the extracted epochs. This method is based on linear regression and reliably removes electrooculogram activity from the EEG signal (Schlögl et al., 2007). Three participants were excluded due to the low number of good trials left after EEG-data preprocessing (<1/3 of the trials).

For each participant, separate ERP averages were computed for each sensor in each of the following conditions: standard, easy target, difficult target and novel.

Based on previous research (e.g., Begleiter et al., 1984; Venables et al., 2011; Gilmore et al., 2012; Venables and Patrick, 2014) and on visual inspection of the current dataset, mean ERP amplitudes were extracted in the time window between 280 and 550 ms from a representative fronto-central cluster (EGI sensors: 6, 7, 15, 16, 23, 24, 30, 207 and 215) to examine the P3a, and from a representative centro-parietal cluster (EGI sensors: 86, 87, 88, 98, 99, 100, 101, 109, 110, 118, 119, 127, 128, 129, 140, 141, 142, 152, 153 and 162) to examine the P3b.

### Statistical Analyses

To test for potential side effects induced by the stimulation, *t*-tests for the ratings comparing tVNS and sham stimulation for each reported subjective symptom were performed, separately. To test the effects of stimulation on autonomic reactivity and salivary levels, a repeated measures analysis of variance (ANOVA) with the within-subject factors *time* (pre vs. post) and *stimulation* (tVNS vs. Sham) was performed for each variable, separately. The effects of tVNS on behavioral performance were assessed for the accuracy and response time (RT), using repeated measures ANOVAs including the within-subject factors *target stimulus* (Target Easy vs. Target Difficult) and s*timulation* (tVNS vs. Sham). For the analyses, errors, defined as those trials with incorrect responses or with RTs below 150 ms (i.e., anticipatory responses) or above 1500 ms (i.e., misses), were discarded (9.7% of trials). To evaluate the effects of tVNS on the brain dynamics of target processing, repeated-measures ANOVAs were carried out with the within-subject factors* stimulus type* (Target Easy vs. Target Difficult vs. Standard) and *stimulation* (tVNS vs. Sham) for frontal and parietal electrode clusters. For these analyses errors were discarded (see above). Similarly, to investigate the effects of tVNS on novelty processing, repeated measures ANOVAs involving the within-subject factors *stimulus type* (Novel vs. Standard) and *stimulation* (tVNS vs. Sham) were conducted for the frontal and parietal sensor clusters. For effects involving repeated measures, assumptions of all statistical tests were checked, and none of these assumptions (i.e., sphericity, outliers and normality of errors) were violated.

To test the relationship between the changes in sAA levels and P3b amplitudes, we ran repeated measures correlation analysis (Bakdash and Marusich, 2017) between the sAA changes and the P3b amplitudes for the easy and difficult targets, separately. Moreover, to test the relation between the tVNS changes in P3b amplitudes and sAA levels, zero-order correlations were performed between the increase of P3b amplitudes (tVNS vs. sham) for easy and difficult targets, and the increase in sAA levels (pre vs. post stimulation). Furthermore, the relationship between the increase in P3a amplitudes increase in sAA was also analyzed. The statistical analyses were conducted using IBM SPSS Statistics 24, JMP 5.0 and R 3.4.3.

## Results

### Subjective Ratings

In general, subjective ratings indicated that the side effects of the stimulation were minimal (*N* = 20; *M* = 1.72, *SD* = 0.73; see Table 1). T-comparisons showed no differences between stimulation conditions (*p*s > 0.203), except for the sensory experience of the stimulation, with higher ratings in the tVNS condition (stinging sensation under the electrodes: *t*_(19)_ = 1.89, *p* = 0.072, *d* = 0.42; skin irritation in the ear:* t*_(19)_ = 3.32, *p* = 0.004, *d* = 0.76), compared to sham. These results indicate that no unpleasant side-effects were experienced in either of the two conditions.

**Table 1:** Mean subjective ratings (standard deviation) for the stimulation side effects in the active and sham condition.

	tVNS	Sham
Headache	1.5 (1.5)	1.85 (1.18)
Nausea	1.2 (0.61)	1.23 (0.55)
Dizziness	1.55 (0.6)	1.7 (1.13)
Neck pain	1.5 (0.2)	1.3 (0.47)
Neck contraction	1.6 (0.95)	1.7 (0.93)
Stinging sensation	2.87 (2.07)	1.95 (1.39)
Ear irritation	1.8 (1)*	1.15 (0.49)
Concentration	3.55 (1.73)	3.45 (1.7)
Fluctuation of feelings	1.65 (0.88)	1.37 (0.81)
Unpleasant feelings	1.94 (1.13)	1.65 (1.04)

N = 20; *p < 0.05.

### Autonomic Results

Results from the cardiovascular and salivary data are presented in Table 2. Heart rate and blood pressure analyses included 20 participants, and sAA level analysis included 18 participants (see “Autonomic Measures” section). A main effect of *time* for heart rate *F*_(1,19)_ = 43.76, *p* < 0.001, $\eta_{\text{p}}^{2}$ = 0.69, and systolic blood pressure *F*_(1,19)_ = 5.0, *p* = 0.037, $\eta_{\text{p}}^{2}$ = 0.21, indicated habituation during the experiment. This reduction was not observed for diastolic blood pressure, *F*_(1,19)_ = 1.87, *p* = 0.186, $\eta_{\text{p}}^{2}$ = 0.09. Most importantly, no main effects of condition or interaction were observed (*p*s > 0.26), suggesting that stimulation did not have any effect on these autonomic changes.

**Table 2:** Mean (standard deviation) of the autonomic and salivary measures before and after the stimulation.

	Time	Heart rate (bpm)	Systolic blood pressure (mmHg)	Diastolic blood pressure (mmHg)	Alpha-amylase (μkatal/l)
tVNS	Pre	78 (12.61)	111.75 (12.48)	71.5 (5.64)	91.05 (59.52)
	Post	66 (9.75)*	108.3 (9.07)*	73.8 (6.86)	140.62 (94.21)*
Sham	Pre	75.4 (14.1)	112.3 (7.51)	73 (8.17)	98 (68.37)
	Post	66 (9.31)*	108.8 (7.44)*	73 (6.57)	117.9 (72.8)

For heart rate, and blood pressure, N = 20; for alpha-amylase, N = 18. *p < 0.05.

For sAA levels, a main effect of *time* was observed, *F*_(1,17)_ = 9.93, *p* = 0.006, $\eta_{\text{p}}^{2}$ = 0.37, reflecting an increase of alpha-amylase during task performance. No main effect of *stimulation*, *F*_(1,17)_ = 2.22, *p* = 0.154, $\eta_{\text{p}}^{2}$ = 0.12, or interaction, *F*_(1,17)_ = 4.0, *p* = 0.062, $\eta_{\text{p}}^{2}$ = 0.19 was found. Subsequent analyses showed, however, that alpha amylase levels significantly increased following the tVNS, (*t*_(17)_ = 3.77, *p* = 0.002, *d* = 0.89) but not following sham stimulation (*t*_(17)_ = 1.47, *p* = 0.158, *d* = 0.35). This finding indicates that tVNS, to some extent, enhanced the activation of the noradrenergic system.

### Behavioral Results

Results from the behavioral performance (*N* = 20) in the visual oddball task are presented in Table 3. Results for performance accuracy (PA) indicated that participants were more accurate detecting easy compared to difficult targets (*F*_(1,19)_ = 21.81, *p* < 0.001, $\eta_{\text{p}}^{2}$ = 0.53). No effects of *stimulation* (*F*_(1,19)_ = 1.41, *p =* 0.249, $\eta_{\text{p}}^{2}$ = 0.07) or interaction were observed (*F* < 1). For RT, participants were also faster during the easy compared to the difficult condition (*F*_(1,19)_ = 129.6, *p* < 0.001, $\eta_{\text{p}}^{2}$ = 0.87), but no effects of *stimulation* or interaction were observed (*F*s < 1).

**Table 3:** Mean (standard deviation) response times (RT) in ms and performance accuracy (PA) for the stimulation conditions transcutaneous vagus nerve stimulation (tVNS) and Sham for easy and difficult Targets.

	Stimulation	Easy	Difficult
RT	tVNS	716 (113.95)	917 (136.79)
	Sham	701 (87.7)	881 (165.15)
PA	tVNS	0.94 (0.078)	0.89 (0.12)
	Sham	0.93 (0.13)	0.86 (0.15)

*N = 20*.

### ERP Results

**Figure 1:**
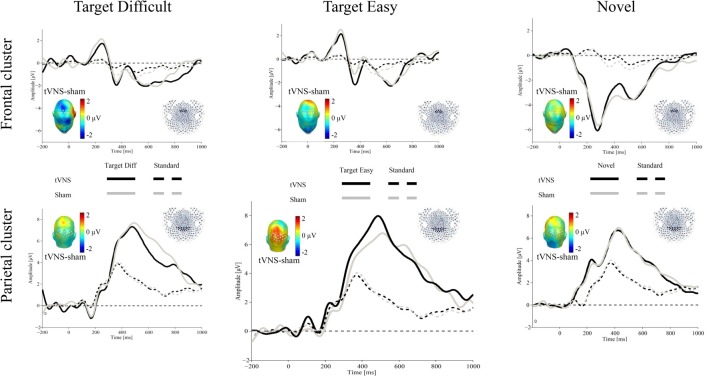
Grand average event-related potentials (ERPs) evoked by target difficult (left), target easy (middle) and novel (right) stimulus (thick lines) and standard stimulus (dotted lines) for the transcutaneous vagus nerve stimulation (tVNS; black) and sham (gray) conditions (*N* = 17). The waveforms represent the ERPs averaged across electrodes within a frontal cluster (upper part) and a central-parietal sensor cluster (lower part) to extract the P3a and P3b, respectively. The scalp topographies of the ERP difference between both conditions are plotted in the inset.

Figure 1 shows the ERPs for all conditions over representative frontal and parietal clusters averaged for 17 participants (see “Electrophysiological Recording” section).

#### Target Stimuli

##### P3a

At frontal areas, results indicated that *stimulus type*, or *stimulation*, did not modulate the P3a amplitude for the target stimuli (*F*s < 1.01).

##### P3b

Over parietal regions, results revealed a main effect of *stimulus type*, *F*_(2,32)_ = 25.89, *p* < 0.001, $\eta_{\text{p}}^{2}$ = 0.61. As expected, larger P300 amplitudes were observed for the targets compared to the standard events. There was no main effect of *stimulation* (*F* < 1), or interaction *stimulus type × stimulation*, *F*_(2,32)_ = 2.41, *p* = 0.106, $\eta_{\text{p}}^{2}$ = 0.13.

To explore the relationship between tVNS and P3b, subsequent exploratory analyses were carried out, using repeated-measures ANOVAs for each target stimuli separately with the within-subject factors *stimulus type* (Target vs. Standard) and *stimulation* (tVNS vs. Sham). For the difficult target stimulus, results showed a main effect of *stimulus type*, *F*_(1,16)_ = 33.55, *p* < 0.001, $\eta_{\text{p}}^{2}$ = 0.67, but no *stimulation* or interaction effects (all *F*s < 1). For the easy target stimulus, the expected effect of *stimulus type* was also observed, *F*_(1,16)_ = 56.49, *p* < 0.001, $\eta_{\text{p}}^{2}$ = 0.78, in the absence of a *stimulation* effect, *F*_(1,16)_ = 2.02, *p* = 0.175, $\eta_{\text{p}}^{2}$ = 0.11. Critically, the interaction stimulus type × stimulation was significant, *F*_(1,16)_ = 4.5, *p* = 0.05, $\eta_{\text{p}}^{2}$ = 0.22. *Post hoc t*-tests showed that whereas no effects of stimulation were observed for P3b amplitudes in response to difficult targets or standard stimuli, *t*s < 1), tVNS, compared to sham, increased P3b amplitudes for easy targets (*t*_(16)_ = 2.04, *p* = 0.058, *d* = 0.49). Although the effects were not two-tailed significant, they followed the predicted direction.

##### Novel Stimuli

To investigate the effects of tVNS on novelty processing, 2-way repeated measures ANOVAs involving the within-subject factors s*timulus type* (Novel vs. Standard) and s*timulation* (tVNS vs. Sham) were conducted for the frontal and parietal sensor clusters.

###### P3a

For P3a, results revealed a main effect of *stimulus type, F*_(1,16)_ = 33.98, *p* < 0.001, $\eta_{\text{p}}^{2}$ = 0.68, indicating larger amplitudes for the standard stimuli compared to the novel ones. However, no effect of *stimulation* or interaction was observed (all *F*s < 1)^2^.

###### P3b

For P3b, a main effect of *stimulus type* was observed, *F*_(1,16)_ = 58.59, *p* < 0.001, $\eta_{\text{p}}^{2}$ = 0.78, as shown by larger activity for the novel, compared to standard stimuli. As for frontal regions, neither the *stimulation* effect nor the interaction reached significance (all *F*s < 1).

##### Association Between P3 Amplitudes and Alpha Amylase Levels

**Figure 2:**
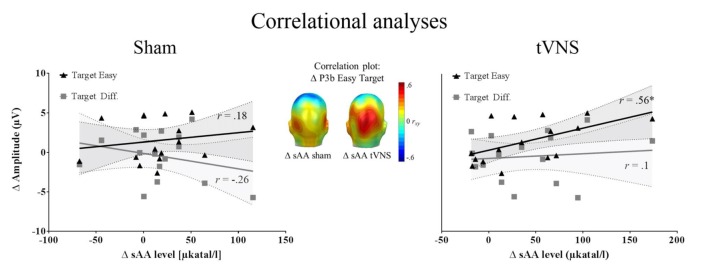
Correlation analysis (*N* = 16). Left and right: zero-order correlations between the increase P3b amplitudes for tVNS compared to sham condition and the increase in salivary alpha-amylase (sAA) during stimulation for sham (left) and tVNS (right) conditions. The target easy condition is represented in black and the target difficult condition in dark gray. Shaded areas represent the confidence intervals of the correlations. Middle: correlation of the above mentioned variables for the easy target condition, across electrodes, showing that the highest correlation matches spatially with the P3b location.

Correlation analysis was performed for 16 participants (see “Autonomic Measures” and “Electrophysiological Recording” sections). The repeated measures correlation analysis revealed that the increase in sAA levels correlated positively with the P3b amplitudes for easy targets across conditions, *r*_(15)_ = 0.50, *p* = 0.04, *r*^2^ = 0.25, whereas this association was not found significant for difficult targets, *r*_(15)_ = 0.17, *p* = 0.5, *r*^2^ = 0.028. Most interestingly, the increase of sAA (post vs. pre) during tVNS was positively correlated with the enlarged P3b amplitudes during tVNS compared to sham stimulation for the easy target condition (*r*_(15)_ = 0.56, *p* = 0.025, *r*^2^ = 0.31), but not for the difficult one (*r*_(15)_ = 0.1, *p* = 0.702, *r*^2^ = 0.001). During sham stimulation, the increase of sAA levels was not related to the P3b enhancement for targets (Easy: *r*_(15)_ = 0.18, *p* = 0.493, *r*^2^ = 0.032; Difficult: *r*_(15)_ = −0.26, *p* = 0.314, *r*^2^ = 0.067; see Figure 2). No relation was observed between the increase in sAA and the increase in P3a amplitudes (−0.34 < *r*s < 0.36, all *p*s > 0.132).

To summarize, we observed a positive relationship between changes in sAA levels and P3b amplitudes for easy targets. When the increase in P3 amplitudes (tVNS vs. sham) was considered, sAA changes showed a positive relation specifically during vagal stimulation. The results point toward a potential association between noradrenergic activation and the P3b, which may be particularly prevalent under continuous vagal stimulation.

## Discussion

In the present study, we investigated the impact of tVNS on the attention-related oddball P300. In addition, changes on sAA levels, as an indirect marker of noradrenergic activation (Warren et al., 2017), following tVNS were also assessed. Although, main analyses did not show significant differential effects of stimulation on P3b amplitudes and sAA levels, direct hypothesis-driven analyses revealed that vagal, compared to sham stimulation specifically increased the amplitude of the P3b, particularly in response to easy target stimuli in comparison to standard stimuli. Vagal stimulation, however, did not modulate the P3b amplitudes to difficult targets and novel stimuli. *Post hoc* analyses also revealed that tVNS, but not sham stimulation, increased sAA compared to baseline, possibly indicating a potentiation of NE release. Changes in sAA levels correlated positively with the P3b amplitudes for easy targets, independently of condition. Furthermore, when the increase in P3b amplitudes (tVNS vs. sham) was considered, sAA changes showed a positive relation, specifically during vagal stimulation. These results point toward an association of the noradrenergic transmitter system in the brain with the P3b that may be particularly prevalent when a systematic activation of the noradrenergic system is carried out via continuous tVNS.

The present results may support the LC-P3 hypothesis (Nieuwenhuis et al., 2005; see also Polich, 2007), which assumes that the P3b derives from the phasic response of NE neurons of the LC that project to posterior cortical regions. Based on the *post hoc* results, we found that stimulation of the vagus nerve increased the release of endogenous NE, as reflected by changes in sAA^3^. Given that the LC is the major source of NE in the brain and that previous studies found that the vagus nerve directly innervates the LC (Dorr and Debonnel, 2006; Raedt et al., 2011), it is likely that the observed changes in the P3b amplitude are modulated via this neural pathway. As hypothesized, tVNS did not show any effect on the P3a amplitude, which may be due to stronger influences from a different neural system on this ERP component (Polich, 2007; Marco-Pallarés et al., 2010).

Interestingly, although the P3b was modulated by tVNS in the easy target condition, we did not observe an increased P3b in response to the difficult targets during tVNS. One potential explanation for this result could be that distinct mental processes were engaged in our target conditions. Whereas in the easy target condition, a simple decision (i.e., left or right button) was required, mental rotation of the head was additionally needed in the difficult target condition. Mental rotation depends upon spatial working memory (Courtney et al., 1998; Courtney, 2004) and comes with costs (Cooper and Shepard, 1973), as reflected by increased reaction times, and decreased PA (see behavioral data). Mental rotation is also associated with activity in the superior parietal lobe, producing a larger ERP negativity over central-parietal regions from 350 ms to 800 ms after stimulus onset (Peronnet and Farah, 1989; Riecanský and Jagla, 2008). Given that the activity generated by mental rotation overlaps with the spatio-temporal characteristics of the P3b, it could be that the enhanced positive-going waveform generated by the difficult targets results from the combination of the P3 response elicited by the target property of the stimulus and the brain activation produced by mental rotation, thus, concealing the enhancing effect of the tVNS on the P3b. Previous fMRI data by Weiss et al. (2009) suggested that this interference may be reduced, to some extent, by certain instructions. Weiss et al. (2009) examined the neural substrates of mental rotation when participants were explicitly instructed to rotate alphanumeric stimuli vs. when participants were instructed to make a discrimination task. The authors observed that the typical fronto-parietal activation produced by mental rotation was only observed when the instruction was explicitly given. With regard to our study, this may indicate that using instructions that do not promote mental rotation (e.g., instructing participants to give a response when a head is presented instead of to indicate the location of the ear on the head), the enhanced effects of tVNS on the P3b we observed for the easy condition may also be observed in the difficult condition. Future studies, however, need to confirm this hypothesis.

Even though our results were mainly based on hypothesis driven *post hoc* testing, we found an indication that, under certain experimental conditions, tVNS influenced the attention-related P3b, plausibly via LC-NE activation, which points towards a promising direction to modulate various cognitive and affective functions via tVNS (Van Leusden et al., 2015). The LC has widespread projections to different brain regions (for a review see Sara and Bouret, 2012) including the hippocampus (e.g., Harley, 2007; Mello-Carpes and Izquierdo, 2013), amygdala (e.g., Williams et al., 1998, 2000; Chen and Sara, 2007; for a review see McIntyre et al., 2012), and frontal cortex (e.g., Clayton et al., 2004; for a review see Arnsten et al., 2012). Through these afferent projections the arousal-modulated LC-NE system is able to facilitate sensory processing (Aston-Jones and Cohen, 2005; Jepma and Nieuwenhuis, 2011), attention (Bouret and Sara, 2005; Corbetta et al., 2008), cognitive flexibility (Aston-Jones and Cohen, 2005; Bouret and Sara, 2005), learning (Aston-Jones et al., 1994; Bouret and Sara, 2004; Bouret and Richmond, 2009), and memory consolidation (Williams et al., 2000; for review, see Mather et al., 2016; McIntyre et al., 2012). TVNS, via activation of the LC-NE system, may facilitate all of these processes. Some studies already showed improvements in emotion recognition (Colzato et al., 2017) cognitive control (Sellaro et al., 2015; Steenbergen et al., 2015; Beste et al., 2016), adaptability (Fischer et al., 2018), flow experience (Colzato et al., 2018), declarative fear extinction (Burger et al., 2016), and associative memory (Jacobs et al., 2015) following transcutaneous vagus stimulation.

Due to its effects on affective and cognitive functioning, tVNS could also be of special relevance for clinical research. Several studies have shown that the P3b amplitude is reduced in different mental disorders including, schizophrenia (e.g., Pfefferbaum et al., 1989), mood disorders (e.g., Bruder et al., 2009; Rongrong et al., 2015; Lang et al., 2017), or anxiety disorders (e.g., Li et al., 2015; Lang et al., 2017). This suggests that the P3b amplitude increase through vagus nerve stimulation may reflect symptomatology improvement in patients diagnosed with these disorders. Substantiating this view, one study using implanted stimulators showed that the enhancing effects of vagus nerve stimulation (VNS) on the P3b amplitudes were correlated with symptom reduction in depression. In a small sample of depressed patients (*N* = 13), Neuhaus et al. (2007) observed that only those who showed an increased P3b amplitude following VNS therapy also showed a decrease in symptom severity. Whether non-invasive tVNS, which is a safe and easy-to-apply method, is likewise effective (for a review see Daban et al., 2008) in reducing symptom severity (Hein et al., 2013; Rong et al., 2016) concomitant with changes in brain activation remains to be seen. Together with these clinical findings, our results suggest that tVNS could be a promising tool to support treatment of mental disorders.

Although the reported side-effects were minimal, participants felt more skin irritation in the ear and stinging sensation under the electrode during tVNS, compared to sham stimulation. One possible explanation for the enhanced sensory experience during tVNS could be the existence of more sensitive-related nerve terminations in the cymba conchae than in the ear lobe, making this area more prone to stimulation sensations (Ellrich, 2011). Importantly, these subjective feelings were not related to the tVNS effects observed in P3b amplitudes and sAA levels, as indicated by nonsignificant correlation between reported feelings and both variables (for P3b amplitude increase to targets: 0.36 > *r*s > −0.26, all *p*s > 0.16; for changes in sAA: (0.04 > *r*s > −0.26, all *p*s > 0.28).

Finally, some limitations of the current study should be mentioned. First, our study sample was relatively small and mainly consisted of female participants. Although prior tVNS studies did not report any sex differences when tested in the context of cognitive control, emotion recognition, or associative memory (Jacobs et al., 2015; Sellaro et al., 2015; Steenbergen et al., 2015; Beste et al., 2016; Colzato et al., 2017) it is possible that female participants are more sensitive to tVNS induced LC-NE activation based on animal work (for a review see Bangasser et al., 2016). In terms of the LC structure, along with larger size, female rates showed more complex dendritic trees than their male counterparts, which could lead to an increase in afferent information coming from the NST, among other afferent pathways (discussed in Bangasser et al., 2016). In terms of the modulatory activation of the LC-NE system, it has been observed that estrogen release influences the synthesis and degradation of NE and this is higher in female rats (e.g., Vathy and Etgen, 1988). Moreover, the modulation of the NE levels seems to be influenced by the rat estrous cycle (Selmanoff et al., 1976). Whether these animal results can be translated to humans, however, remains unclear. Future studies need to test whether tVNS is particularly affecting female than male participants.

To summarize, we found indication for a modulatory influence of tVNS on the P3b to easy targets compared to standard stimuli, which may be mediated by activation of the noradrenergic system, as assessed with sAA level changes. Due to the small sample in the current study, however, additional research in this field is clearly warranted.

## Conclusion

We found that tVNS produced larger P3b amplitudes to easy targets, relative to standards and increased sAA levels compared to baseline (based on *post hoc* pre vs. post comparison). Given that the P3b was associated with stronger sAA activity, our findings indicate that, at least under low cognitive load, the P3b is modulated by tVNS likely via stimulation of the noradrenergic system. In light of the existent relationship between diminished P300 activity and vulnerability to distinct psychopathologies, the present results also may give some insight for the use of tVNS to clinical research as a promising tool to support treatment of mental disorders.

## Author Contributions

CV-B and MW conceived the presented idea. All authors discussed the design. CV-B programed the experiment and analyzed the data. All authors discussed the results and contributed to the final manuscript.

## Conflict of Interest Statement

The authors declare that the research was conducted in the absence of any commercial or financial relationships that could be construed as a potential conflict of interest.

## Footnotes

^1^http://www.labservice-dresden.de

^2^At first glance, a larger P3a for standard stimuli compared to novel events seems contradictory to the previous literature. The inspection of the waveforms, however, suggested that the decreased P3a for novel stimuli was due to a larger N2 component preceding the P3a. To better isolate the P3a, we recalculated its mean amplitude, using a peak-to-peak analysis, calculating the difference between the P3a mean amplitude (280–550 ms) and the N2 mean amplitude (250–280 ms). Results showed a main effect of *Stimulus Type, F*_(1,16)_ = 139.26, *p* < 0.001, $\eta_{\text{p}}^{2}$ = 0.89, but not effect of *Stimulation* or interaction, (all *F*s < 2.39, *p*s > 0.14, $\eta_{\text{p}}^{2}$ < 0.13). However, in this case, the Novelty stimuli showed larger amplitude (*M* = 5.58 μV) than the standard ones (*M* = −0.029 μV).

^3^tVNS was associated with an increase of sAA levels, but not with changes in heart rate or blood pressure. sAA has been suggested as an indirect index of noradrenergic activity in the central nervous system (Chatterton et al., 1996; Warren et al., 2017) indicating that, at least to some extent, tVNS facilitated the noradrenergic release in the brain. tVNS, however, did not modulate indicators of tonic sympathetic activation, such as systolic blood pressure or heart rate. This is in line with previous findings (Clancy et al., 2014; Sellaro et al., 2015; Colzato et al., 2017; De Couck et al., 2017). In a recent study, Clancy et al. (2014) observed that tVNS increased heart rate variability, an indicator of parasympathetic activity, and decreased tonic muscle sympathetic nerve activity, suggesting that tVNS may exert special influence on the tonic activation of the parasympathetic nervous system, and, to some extent, on the reduction of the tonic sympathetic nervous system. This finding was partly replicated in another study (De Couck et al., 2017). It must be noted, however, that the autonomic responses measured in this and prior studies reflected tonic rather than phasic, stimulus-related responses. Most likely, the increase in arousal activation produced by tVNS is better observed at a phasic level. In this sense, our electrophysiological findings may give support to this view. The P3b has recently been proposed as a brain indicator of the orienting response generated by the presence of relevant novel events (Nieuwenhuis et al., 2011). Along with the P3b, the orienting response is associated with peripheral physiological changes related to phasic activation of the sympathetic system such as the heart rate, the skin galvanic response, and the pupil dilation. Thus, it could be that the enhanced arousal activation produced by tVNS may be particularly reflected in the autonomic phasic responses. To elucidate this, future studies combining both phasic central and peripheral responses would be needed.
